# Safety and Efficacy of Spray Intranasal Live Attenuated Influenza Vaccine: Systematic Review and Meta-Analysis

**DOI:** 10.3390/vaccines9090998

**Published:** 2021-09-07

**Authors:** Giulia Perego, Giacomo Pietro Vigezzi, Giulia Cocciolo, Federica Chiappa, Stefano Salvati, Federica Balzarini, Anna Odone, Carlo Signorelli, Vincenza Gianfredi

**Affiliations:** 1 School of Medicine, Vita-Salute San Raffaele University, Via Olgettina, 58, 20132 Milan, Italy; perego.giulia@hsr.it (G.P.); vigezzi.giacomopietro@hsr.it (G.P.V.); cocciolo.giulia@hsr.it (G.C.); chiappa.federica@hsr.it (F.C.); salvati.stefano@hsr.it (S.S.); signorelli.carlo@hsr.it (C.S.); 2 Dipartimento per la Programmazione, Accreditamento, Acquisto delle Prestazioni Sanitarie e Sociosanitarie (PAAPSS), Servizio Autorizzazione e Accreditamento, Agenzia di Tutela della Salute (ATS) di Bergamo, Via Galliccioli, 4, 24121 Bergamo, Italy; federica.balzarini@ats-bg.it; 3 Department of Public Health, Experimental and Forensic Medicine, University of Pavia, 27100 Pavia, Italy; anna.odone@unipv.it; 4 CAPHRI Care and Public Health Research Institute, Maastricht University, 6200 MD Maastricht, The Netherlands

**Keywords:** intranasal live attenuated influenza vaccine, inactivated influenza vaccine, adult, infant, immunogenicity, immune response, antibody response, safety

## Abstract

Although influenza is a major public health concern, little is known about the use of spray live attenuated influenza vaccine (LAIV) among adults. For this reason, we conducted a systematic review and meta-analysis to investigate the efficacy and safety of LAIV, especially in adults with/without clinical conditions and children <2 years, with the final aim of possibly extending the clinical indications. PubMed/MEDLINE and Scopus were the two databases consulted through February 2021. The Preferred Reporting Items for Systematic Reviews and Meta-Analyses guidelines were followed. A critical appraisal was conducted. Analyses were performed by using ProMeta3 software. Twenty-two studies were included, showing that LAIV was associated with a higher probability of seroconversion when compared with a placebo and considering the A/H1N1 serotype (pooled OR = 2.26 (95% CI = 1.12–4.54), *p*-value = 0.022; based on 488 participants, without heterogeneity (I2 = 0.0%)). The meta-analysis also confirmed no significant association with systemic adverse events. Only rhinorrhea, nasal congestion, and sore throat were significantly associated with LAIV compared to the placebo. Despite limited available evidence, LAIV has proved to be a safe and effective flu vaccination, also due to its very low invasiveness, and our review’s results can be considered a starting point for guiding future research and shaping forthcoming vaccination campaigns.

## 1. Introduction

Influenza is an acute respiratory infection caused by influenza viruses, which belong to the single stranded-RNA genome family of *Orthomyxoviridae*. It is one of the most significant and commonly occurring vaccine-preventable diseases. Thus, it is a significant source of morbidity and mortality worldwide, with an attributable estimate of 54.5 million lower respiratory tract infections (LRTIs) in 2017 [1]. It causes illnesses that range from mild to severe, occasionally requiring hospitalisation and, at times, leading to death. The disease severity may vary according to the seasonal flu viral strain, the specific strains in the vaccine, and patients’ characteristics, such as age, comorbidities, or underlying chronic conditions. As recommended by the World Health Organisation (WHO), annual vaccination is currently the most effective strategy to control seasonal influenza infections [2,3], especially for people at a greater risk of severe disease or complications when infected, i.e., pregnant women, children aged <5 years old, the elderly (>65 years old), or subjects with underlying clinical conditions [4,5]. To reduce their risk of contracting influenza [6] and prevent transmission to susceptible patients [7], influenza vaccination on healthcare workers is strongly encouraged, if not required, in many hospitals. Despite the development and widespread availability of safe and efficient vaccines, vaccination coverage, especially among the most vulnerable populations, is still far from the recommended threshold (at least 75% of the population) [8]. This low coverage is one of the leading causes of the high burden of influenza, with a range of 250,000–645,000 estimated deaths every year from seasonal influenza-associated respiratory complications worldwide [9,10].

Nowadays, there are two types of flu vaccines currently available: inactivated influenza vaccines (IIVs) and live attenuated influenza vaccines (LAIVs). On the one hand, IIV is approved for use in subjects aged six months and older, including persons with underlying chronic medical conditions and pregnant women, and it is administered by intramuscular injection [11]. On the other hand, LAIV, being a live attenuated virus, is approved in the USA for use in healthy individuals between 2 and 49 years [12], and in Europe for individuals between 2 to 18 years [13], and should not be administered to pregnant women [10]. The most significant advantage of LAIV is the non-invasive route of administration by nasal spray. Furthermore, it imitates natural infection, conferring mucosal immunity, and therefore enabling this vaccine to be the most suitable candidate for mass immunisation, especially in pandemics [14].

Despite being primarily designed for children, other categories may also benefit from live attenuated vaccines. LAIV efficacy and its impact on vulnerable groups are still debated. This systematic review aims to investigate whether the LAIV is safe and effective in adults, including those with underlying clinical conditions, pregnant women, and children younger than 24 months.

## 2. Materials and Methods

The Preferred Reporting Items for Systematic Review and Meta-analyses 2020 (PRISMA) guidelines [15] and the Meta-Analysis of Observational Studies in Epidemiology (MOOSE) guidelines [16] were used to guide the reporting and the conduction of this systematic review with meta-analysis.

A study protocol was developed in advance by the research team. It was registered in the PROSPERO database (ID number CRD42021228770), a prospective international registry of systematic reviews funded by the National Institute of Health Research. The research question, search strategy, inclusion and exclusion criteria, primary outcomes, strategy for data extraction, and data synthesis were determined in the protocol. In particular, our research question was if the live-attenuated influenza vaccine, when intranasally administered using a spray, is safe and effective in infants younger than 24 months, adults, patients with comorbidities, and pregnant/breastfeeding women.

### 2.1. Search Strategy and Data Sources

Two electronic databases (PubMed/Medline and Scopus) were searched the same day (6 February 2021) to identify potentially relevant articles. The search strategy, firstly developed in PubMed and then adapted to Scopus, included a specific selection of keywords, such as the MeSH terms and other text words combined with the Boolean operators AND, OR, and NOT. The whole search strategy of both databases is available in Supplementary Materials Table S1. In brief, it was based on four key components: (i) influenza vaccine, (ii) influenza and synonyms, (iii) administration way (nasal and similar), and (iv) study design. Screening of the reference list of included articles and consultation of experts in the field were conducted to identify any additional relevant articles. Lastly, in the case of missing or incomplete data, corresponding authors of included articles were contacted.

### 2.2. Inclusion and Exclusion Criteria

In accordance with the Cochrane Collaboration [17], inclusion/exclusion criteria were detailed based on Population, Intervention, Comparison, Outcome, and Study design (PICOS) [18]. The literature search was limited to English language and human subjects, and no time filter was applied to the research.

In brief, the population of interest was defined as infants younger than 24 months, adults (≥18 years), pregnant/breastfeeding women, and subjects with comorbidities. Only studies that analysed the live-attenuated influenza vaccine administered intranasally as spray were included.

The LAIV vaccine was compared with a placebo and/or other influenza vaccines, such as IIV. No other comparisons, such as different groups or different administration ways of the same vaccine, were considered eligible. Moreover, studies assessing LAIV efficacy or safety among children were unsuitable because existing evidence showed solid and consistent safety and efficacy in this age group [19].

To be included in the systematic qualitative review, papers must report data regarding the LAIV vaccine’s safety, efficacy, and effectiveness. Regarding study design, only observational studies and randomised clinical trials were considered eligible; on the contrary, in vivo studies, in vitro studies, studies not published as peer-reviewed, systematic reviews, meta-analyses, books, book chapters, theses, protocols, and non-full-text papers (abstracts, conference papers, letters, commentaries, errata, corrections, editorials, and notes) were excluded from the review.

We excluded studies regarding children or adolescents, papers assessing influenza vaccines other than LAIV, and articles that analysed outcomes different from the above stated. A more detailed draft of eligibility criteria can be found in Supplementary Materials Table S2.

### 2.3. Quality Assessment

The quality evaluation of papers included in the systematic review was carried out independently by two researchers from the team, according to validated tools, and was revised by another author. Any disagreement was then solved with discussion among the authors; a fourth author was consulted in case of disagreement. A judgment was then assigned individually to each paper. The judgement could be “low” or “high” risk of bias or could express “some concerns”. An independent assessment was produced for both the considered outcomes, efficacy, and safety of the interventions. The included studies were assessed by using the Risk of Bias-2 (RoB-2) [20] of the Cochrane Collaboration tool for randomised trials, the Newcastle-Ottawa Scale (NOS) [21] for observational studies and adapted for cross-sectional studies [22], and the Risk of Bias in Non-Randomised Studies of Interventions (ROBINS-I) [23] for non-randomised studies that compare the health effects of two or more interventions. In detail, the risk of bias assessment with RoB-2 included the following five domains: randomisation process, deviations from intended interventions, missing outcome data, measurement of the outcome, and selection of the reported result. ROBINS-I tool included the following domains: bias due to confounding, in choice of participants, in the classification of interventions, due to deviations from intended interventions, due to missing data, in the measurement of outcomes, and in the selection of the reported results. For cohort studies, the NOS scale evaluated selection, comparability and outcome, the same domains considered in the NOS adapted for cross-sectional studies.

### 2.4. Study Selection and Data Extraction

The selection process was carried out in two steps. The first screening was independently conducted by four authors (F.C., G.C., S.S., and G.P.V.), based on title and abstract, and only eligible articles were then evaluated in full text. As done in previous studies [24,25], data extraction was conducted independently by five authors (F.C., G.C., G.P., S.S., and G.P.V.), using a standardised spreadsheet elaborated by the team in Microsoft Excel^®^ for Windows (Microsoft Corporation, Redmond, Washington, DC, USA) and pre-piloted on five randomly selected papers to increase methodological concordance among authors. Any disagreement was solved with discussion among the authors; if controversy persisted, a sixth author (V.G.) was consulted.

In agreement with previous works [26,27], several qualitative and quantitative data were extracted: author(s) and year, country, study design, study period, sample size, population characteristics, comparison, doses administered, and scheme (as well as the dosage), vaccine composition, outcomes, antibody screening methods, funds, and conflicts of interest.

### 2.5. Outcomes Definition

In our review, we considered as outcomes both efficacy and safety. Regarding efficacy, we used the seroconversion rates, expressed as a 4-fold increase in the antibody titer. Regarding safety, we considered all types of systemic (severe or mild) and local adverse events following immunisation.

### 2.6. Statistical Methods

To contribute to the meta-analysis, articles must report data concerning seroconversion rates (considered as the 4-fold increase in antibody title) to evaluate the efficacy and adverse events ratios to assess safety. We estimated the odds ratio (OR) and corresponding 95% confidence interval (CI) for each study based on the number of events (both for seroconversion and adverse events manifestation in both groups, intervention and control) and total sample size. Consequently, the pooled effect size (ES) was reported as OR. The comparison group was performed between the intervention group, identified as those who received LAIV, and the control group, identified as those who received IIV or placebo. As done before [28,29], we applied a fixed and random model. When the universe of studies is sufficiently similar to those in the study sample or just a few studies included, the fixed model is the appropriate one. In the random effect, model inferences are not limited to the studies of the sample. The universe of studies is likely to represent different characteristics, and generalisations are based on studies that differ from those in the study sample. In this perspective, the random effect model is recommended if heterogeneity estimated values are considered high. The heterogeneity among included studies was evaluated through Chi^2^ and I^2^ tests. Heterogeneity was deemed high when I^2^ values > 75%, moderate when I^2^ values ranging between 50 and 75%, low for values ranging between 25 and 50%, and no heterogeneity for values below 25%. The graphical evaluation of the Funnel plot and the Egger’s regression asymmetry test were used to estimate potential publication bias; statistical significance was set at *p* < 0.10 [30]. If any publication bias was detected, the trim and fill method, searching missing studies to the right of overall, was used to adjust by publication bias [31]. The meta-analysis was performed by using the software Prometa3^®^ (Internovi, Cesena, Italy).

## 3. Results

### 3.1. Literature Search

**Figure 1:**
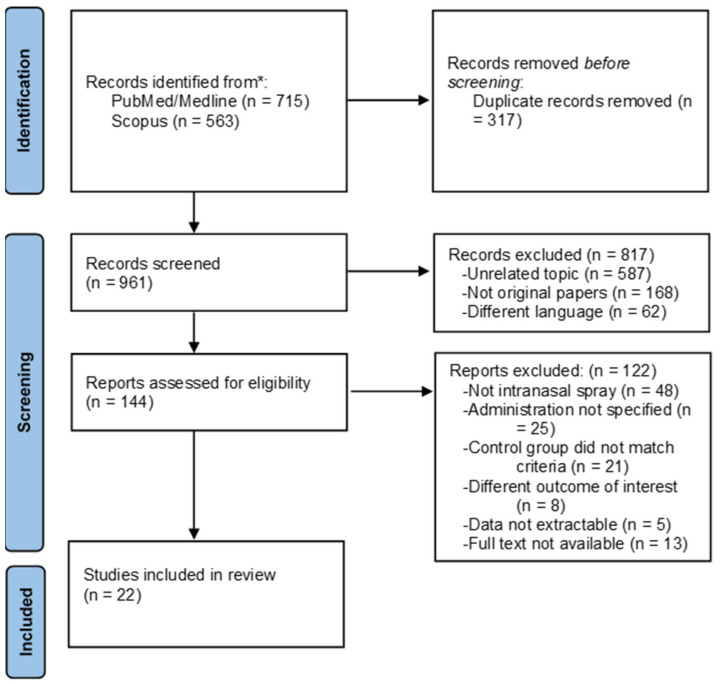
Flow diagram of the selection process.

We retrieved 1278 articles: 715 articles from PubMed and 563 articles from Scopus. After a preliminary screening, 317 duplicates were excluded, 168 were not original papers (e.g., review, letter to the editor, editorial, protocols), 587 articles covered a different topic, and 62 papers were published in other languages. After title and abstract screening, 144 articles were consulted in full at the end of the screening procedure: 22 articles were included in the systematic review [32,33,34,35,36,37,38,39,40,41,42,43,44,45,46,47,48,49,50,51,52,53], whereas 122 articles were excluded with reasons. Reasons of exclusion were mainly because LAIV was not administered by intranasal spray, for instance, via aerosol or drops (n = 48), or the intranasal administration was not specified (n = 25), the control group did not match our inclusion/exclusion criteria, or the study was without comparison (n = 21). Lastly, eight studies had different outcomes of interest (no efficacy neither safety). In five articles, data were not extractable, and, in 13 articles, full text was not available (despite the efforts performed in retrieving them). Figure 1 shows the selection process.

### 3.2. Characteristics of Included Studies

Included studies were distributed over a period that goes from year 1976 [53] to year 2020 [39]; half of them (n = 11, 50%) were published from 2011 until 2020 [32,33,34,35,39,41,43,44,45,46,47], six studies in the previous decade (2000–2010) [37,38,40,48,51,52], and lastly five studies between 1976 and 1999 [36,42,49,50,53] (of which only two articles prior to 1990 [50,53]. Almost all continents were represented in the retrieved studies. Indeed, half of the studies were conducted in the USA (n = 11, 50%) [32,33,34,36,37,38,40,42,48,49,52], followed by Russia (n = 4, 18.2%) [39,45,46,47] and Europe (one study each in Finland, Norway, The Netherlands, and the UK) (n = 4, 18.2%) [41,50,51,53]; two studies (9.1%) were conducted in Asia (Thailand) [43,44]; and only one study in South Africa (4.5%) [35]. No studies were retrieved from Oceania.

In regard to the study design, out of 22 studies, we retrieved two observational studies of which one longitudinal cohort study [52] and one cross-sectional study [48], the remaining 20 (n = 90.9%) studies were all clinical trials, of which 17 (85%) articles specifically reported information regarding randomisation (the others are not specified) [32,33,34,35,36,38,39,40,42,43,44,45,46,47,49,50,51]. Moreover, 13 (65%) of them were double-blind [33,38,39,40,42,43,44,45,46,47,49,50,51], two studies (10%) were open-label [35,37], and the remaining five (25%) did not report any details [32,34,36,41,53].

Nineteen (86.4%) studies referred to a single flu season, while only two (9.1%) studies referred to multiple seasons [33,52]. In one paper, the study period was not specified [38]. Regarding study population and sample size, the vast majority of the included studies recruited adults (n = 20, 90.9%); almost all were healthy adults (n = 17, 85%), whereas one study included breastfeeding women [33], one study included HIV-infected adults [38], and one study included community-dwelling adults in which 90% of the subjects had at least one chronic condition [35]. Lastly, only two studies (9.1%) recruited children: one study population was based on healthy children [51] and the other on partially immunocompromised children with cancer in remission or undergoing chemotherapy [34]. Intervention group sample sizes ranged between 10 subjects [37] and 3041 [42] subjects, with a mean of 323 subjects. For the control group, the sample sizes ranged between 5 subjects [37] and 1520 subjects [42]. However, most intervention-control groups included 10 and 30 subjects.

Referring to the type of intervention and the control group, 12 studies (54.5%) were placebo-controlled [33,38,39,40,42,43,44,45,46,47,49,50,51]; in seven studies (31.8%), LAIV was compared with IIV [33,34,35,37,41,49,52]; in two studies (9%) [36,53], the LAIV spray vaccine was compared with LAIV administered by nasal drops; and lastly, one study [48] compared three groups, namely (1) subjects who received IIV, (2) subjects who received LAIV, and (3) unvaccinated subjects.

With regard to vaccine dose and scheme, two studies did not report information [33,48], nine studies administered only one dose of vaccine (42.9%) [35,36,37,38,41,42,49,52,53], and nine other studies administered two doses (42.9%) [39,40,43,44,45,46,47,50,51], mostly 28 days apart. Lastly, in one study [34], either one or two doses were administered based on the age of the subjects in the cohort. Viral strains included in the vaccine composition were reported in 19 studies (86.4%), whereas, in three studies, this information was missing [32,48,52]. The most frequently analysed viral strain in the vaccine composition was H1N1 (n = 13, 59.1%), followed by H3N2 (n = 11, 50%) and B (n = 8, 36.4%): these three strains were analysed at the same time, administered as a trivalent LAIV in most of the cases (n = 9, 40.9%). Other viral strains were less frequently analysed, such as H7N9, which was taken into account in two Russian studies [39,47]; H5N2, which was analysed in a Russian [46] and a Thai study [44]; and H7N3 and H2N2 were analysed only once, respectively, by Rudenko 2014 [45] and White 1976 [53]. A detailed description of the included studies is reported in Table 1.

### 3.3. Qualitative Assessment of LAIV Efficacy

Among the 22 studies included in the current review, 18 tested the efficacy of the LAIV vaccine (81.8%). The most frequently used laboratory test was the hemagglutination inhibition assay (HAI), which was employed in 17 of the included studies (94.4%), followed by the enzyme-linked immunosorbent assay (ELISA assay), applied in eight out of 18 studies (44.4%) and finally by the microneutralisation test (MN test), which was used in six studies out of 18 (33.3%). Only one study assessed the efficacy estimating the incidence rate of hospitalisation due to pneumonia, influenza, or ILI; and the results showed a higher incident rate among those unvaccinated, followed by those vaccinated with LAIV, whereas the lowest hospitalisation rate was recorded for those vaccinated with IIV [52].

Even when the same laboratory test was carried out, the data were reported differently, evaluating, for instance, the two-fold increase in the antibody titer or the geometric mean titers (GMTs) from pre-vaccination to post-vaccination, thus motivating significant heterogeneity. In the meta-analytical evaluation, we considered a 4-fold rise in the antibody titer, as it was the most common identified measure (Supplementary Materials Table S3).

### 3.4. Qualitative Assessment of LAIV Safety

Among the 22 studies included in the review, 16 examined LAIV safety (72.7%) [32,33,34,35,36,38,39,40,42,43,44,46,48,49,50,51]. Adverse events following immunisation were collected mainly through the use of diary cards or similar (such as symptom cards and checklists, or memory-aid worksheets); 12 of the 16 studies (75%) applied this method. For the remaining four studies, no further information about the collection method or the monitoring for safety data was given [33,35,36,50]. Within the included studies, several different adverse events were considered and investigated, and some were considered only by a few of them (Supplementary Materials Table S4a,b). The meta-analysis focused only on the most frequently inspected symptoms: fever, fatigue, myalgia, headache, cough, sore throat, nasal congestion, and rhinorrhea (Supplementary Materials Table S4a).

### 3.5. Assessment of the Study Quality

With regard to observational studies, Wang et al.’s [52] study was considered to be of high quality, with a score of 9 in the NOS scale (categorised as follows: QS > 7 high quality, 5 < QS ≤ 7 moderate quality, and QS ≤ 5 low quality), while Speroni et al. [48], using the NOS scale adapted for cross-sectional studies, obtained a total score of 6. A detailed quality assessment of observational studies is reported in Supplementary Materials Table S5.

For the quality assessment of randomised and non-randomised intervention trials, the evaluation only allows a quality judgment without quantitative results ranging from high risk of bias to some concerns and low risk of bias. The overall risk of bias for the included studies was considered generally low. However, with reference to randomised intervention studies, the overall judgement for risk of bias was “some concerns” for six studies [32,34,35,38,42,43] out of 17. In detail, some studies were unclear in reporting sections with a description of the randomisation process [43], aroused some doubt about the deviations from the intended interventions [32,34,35,38,42] and with regard to the measurements of the safety outcome [32,34,35,42], potentially introducing selection performance bias as well as detection bias. The risk of bias due to incomplete outcome data and selection of reported results was considered low. Nevertheless, the other included randomised studies were judged as having a low risk of bias [33,36,39,40,44,45,46,47,49,50,51].

Referring to the ROBINS-I tool, overall judgment was of moderate risk of bias for all the non-randomised intervention studies [37,41,53]. In detail, some domains showed a moderate risk of bias due to confounding [37,41,53], in the selection of participants [37,41,53], in the classification of interventions [41,53], due to deviation from intended interventions [53], due to missing data [53], in measurements of outcomes [53], and in the selection of the reported results [53].

**Figure 2:**
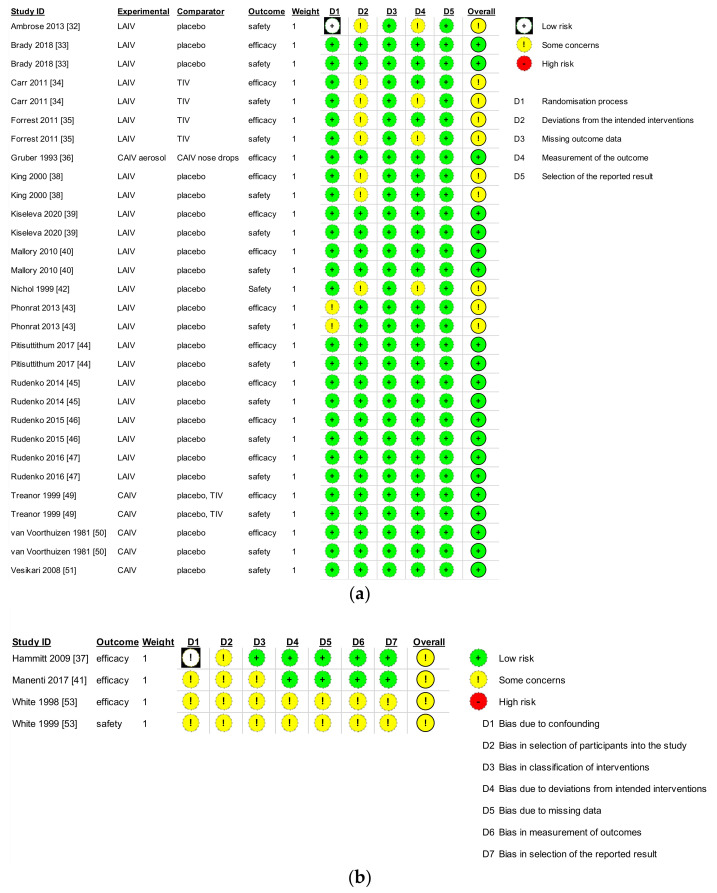
Quality assessment of the included trials, using the Risk of Bias-2 (RoB-2) (**a**) or the Risk of Bias in Non-Randomised Studies of Interventions (ROBINS-I) (**b**) of the Cochrane Collaboration tool, based on study design.

Furthermore, there was an adequate level of detail on the study design, research questions, and aims. A significant limitation for most studies was the small number of subjects enrolled. A summary of these results is depicted in Figure 2a,b.

### 3.6. Meta-Analysis Assessing LAIV Efficacy among Healthy Adults

**Figure 3:**
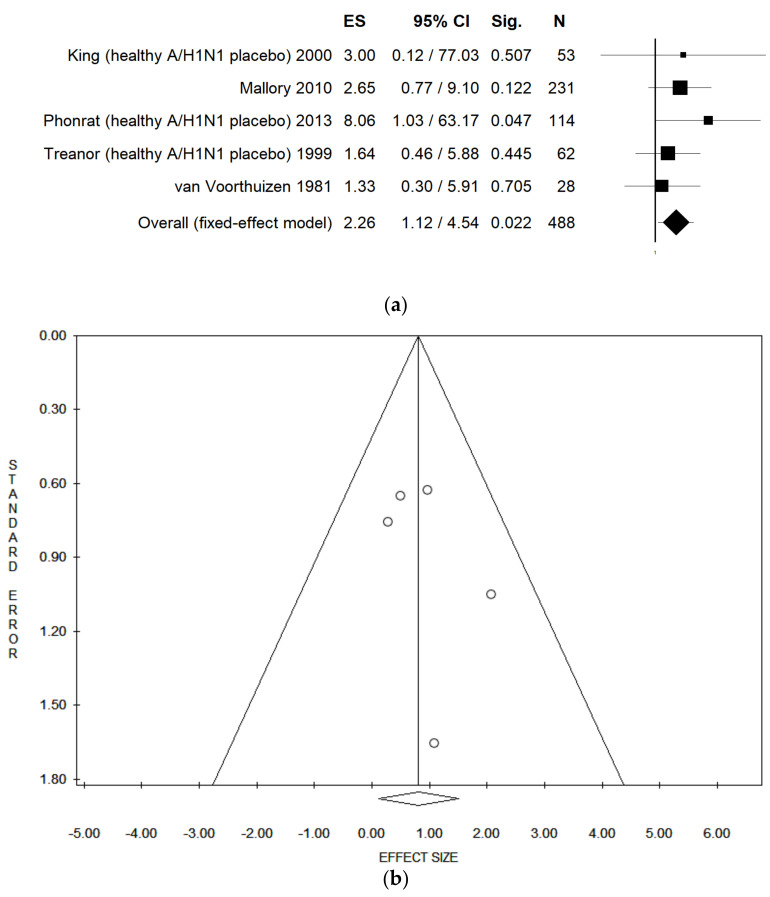
(**a**) Forest plot and (**b**) funnel plot of the meta-analysis comparing seroconversion of LAIV compared placebo of healthy adults for A/H1N1. ES: effect size reported as odds ratio. Sig: *p*-value. References: King 2000 [38], Mallory 2010 [40], Phonrat 2013 [43], Treanor 1999 [49], van Voorthuizen 1981 [50].

When pooling data in a meta-analysis, LAIV was associated with a higher probability of seroconversion when compared with placebo and considering the A/H1N1 serotype among healthy adults (pooled OR = 2.26 (95% CI = 1.12–4.54), *p*-value = 0.022; in both fixed and random effect model; based on 488 participants, with no statistical heterogeneity (Chi2 = 2.28, df = 4, I2 = 0.0%, *p*-value = 0.684)) (Figure 3a). No publication bias was found, both considering fixed- and random-effect model, as demonstrated by the symmetry of the funnel plot and confirmed by Egger’s linear regression test (intercept 1.04, t = 0.89, *p =* 0.440) (Figure 3b). The statistically significant association was not confirmed when studies comparing LAIV with placebo and IIV in healthy adults were combined altogether. In this case, in the fixed-effect model, pooled OR = 1.01 (95% CI = 0.54–1.90) and *p*-value = 0.973; in the random-effect model, pooled OR = 1.16 (95% CI = 0.23–5.78) and *p*-value = 0.854; both based on 551 participants, with high statistical heterogeneity (Chi2 = 29.56, df = 5, I2 = 83.08%, *p*-value = 0.000).

Considering the A/H3N2 strain, among studies assessing the seroconversion of LAIV in healthy adults compared with placebo [38,49] or compared with IIV [38,49], only two studies for each comparison were retrieved, and for this reason, it was not possible to carry out a meta-analysis.

### 3.7. Meta-Analysis Assessing LAIV Efficacy among Immunocompromised Subjects

Due to the low number of studies conducted among specific subgroups of immunocompromised subjects, in this subgroup analysis, we combined HIV-infected subjects, immunocompromised cancer patients, subjects older than 65 years, and pregnant or breastfeeding women. We performed a separate meta-analysis for each serotype assessed in primary studies (A/H1N1, A/H3N2, and B). Moreover, we also considered only studies comparing LAIV with IIV, and, in a separate sensitivity analysis, we combined studies comparing LAIV with IIV and LAIV with placebo. In all of these analyses, results showed a statistically significant lower probability of seroconversion for the intervention group (with LAIV) compared to the control group (both only considering IIV or combining IIV and placebo). Results are shown in Table 2.

### 3.8. Meta-Analysis Assessing LAIV Safety

Due to the high heterogeneity in adverse events following immunisation assessed in the included studies, we performed several separate meta-analyses based on the symptoms reported. However, due to the high heterogeneity and the generally low number of studies focusing on LAIV vs. IIV, it was possible to assess the risk of adverse events only for studies comparing LAIV with placebo and only in healthy adults, except for fever and cough, which were also explored among immunocompromised subjects (in this case, we pooled studies including HIV-infected subjects, immunocompromised cancer patients, and newborn younger than 24 weeks).

When pooling data regarding healthy adults receiving LAIV, none of the analysed symptoms showed a higher risk of events compared to subjects who received placebo (data are shown in Table 3), other than local symptoms, such as sore throat (only when the fixed-effect model was applied, OR = 1.74 (95% CI = 1.43–2.13), *p*-value = 0.000), nasal congestion (OR = 2.33 (95% CI = 1.34–4.04), *p*-value = 0.003, in both fixed and random effect model), and rhinorrhea (only when the fixed-effect model was applied, OR = 2.37 (95% CI = 1.99–2.83), *p*-value = 0.000). No publication bias was found in none of the performed analyses (data are shown in Table 3).

## 4. Discussion

To the best of our knowledge, this is the first systematic review with meta-analysis that specifically assessed both the efficacy and safety of a LAIV intranasally administered via spray. In total, we identified 22 studies, of which 18 assessed LAIV efficacy and 16 LAIV safety (the sum is higher than the total because some studies assessed both efficacy and safety). In particular, focusing on efficacy (as a 4-fold increase in antibody titer), our results showed a high probability of seroconversion after administration of the LAIV intranasally spray when compared against the placebo, but particularly for A/H1N1 serotype and only referring to healthy adults. Indeed, this finding was not confirmed when another serotype—for instance, A/H3N2—was considered. Nevertheless, in the latter analysis, only four studies were retrieved [36,38,49,53], and for this reason, caution is needed in the interpretation of data. Moreover, a smaller sample size was reached in this case, and the wide confidence interval might be explained because of this statistical element. Meanwhile, only two studies [35,38] assessed the efficacy of LAIV intranasally spray compared to placebo in groups different from healthy adults, in particular, people with HIV [38] and the elderly [35]. In this case, no conclusions can be drawn due to the differences in subjects’ characteristics and the paucity of the studies; however, the two studies both found a lower probability of seroconversion in those subjects with LAIV compared to a placebo. Similar and predictable results were also found in studies assessing the efficacy of LAIV in comparison with IIV in subjects with comorbidities, which showed a lower probability of seroconversion among those who received LAIV, regardless of the virus serotype analysed.

Concerning the safety of LAIV, all the included studies compared LAIV vs. placebo, and all the results supported a very high level of safety since most of the assessed symptoms did not differ between the two groups (fever and cough in both healthy and immunocompromised subjects, fatigue/tiredness, myalgia, and headache only in healthy adults). Only local symptoms, such as sore throat, nasal congestion, and rhinorrhea, showed a significantly higher rate among the intervention group than the placebo, mainly in fixed-effect models.

Results of this review highlighted a critical gap in knowledge. In particular, we failed to identify randomised control studies involving vulnerable subjects. Indeed, in our meta-analysis, we combined simultaneously breastfeeding women, immunocompromised patients because of cancer or HIV, and the elderly. No studies were conducted on healthcare workers, also considered at higher risk of influenza because of professional exposure. At the same time, an age-stratified analysis was not possible because only two studies were conducted in subjects older than 65 years, and none of the retrieved studies was conducted in subjects younger than two years of age.

Regarding the geographic distribution of the studies, almost all countries were well covered (America, Asia, Europe, and Africa). However, the highest number of studies was conducted in America, whereas the lowest was in Africa, highlighting a disequilibrium between developed and developing countries.

Considering the study design, almost all included studies were trials, but two were observational; however, the quality of included studies was quite good. The overall risk of bias was judged low or arising moderate concern for all the included studies: no severe or critical risk of bias was identified in any domain of the assessments. This generally medium/high quality of included studies allowed us to be confident about results obtained in our meta-analysis.

Generally speaking, the results of our review should be taken with caution because we did not assess the matching between serotypes contained in administered vaccines and circulating serotypes in the respective influenza season. Moreover, in most cases, studies did not verify the antibody titer before subjects’ allocation in the intervention or control group.

Indeed, influenza prevention is still a major public health concern, not only as a result of low vaccination rates but also due to intrinsic characteristics of the vaccines available and the virus itself. Characteristics of the vaccine are one of the main critical aspects, as proven by the low effectiveness of LAIV from 2013 through 2016 seasons. The Centers for Disease Control and Prevention (CDC) Advisory Committee on Immunisation Practices (ACIP) voted down the use of LAIV for the 2016/2017 flu season [54]. The instability of the vaccine was speculated to have caused the reduced efficacy, which could also contribute to the safety outcome. However, after a 2-year absence, the LAIV vaccine was reintroduced in the 2018/2019 influenza vaccine schedule. This new decision was taken based on additional studies performed, according to which no statistical differences were detected between LAIV and IIV efficacy [55]. Regarding the characteristics of the virus itself, random genetic mutations constantly occur in the genome while the influenza virus replicates in a cell. These alterations can lead to changes in the virus’s surface proteins, the HA (hemagglutinin) and NA (neuraminidase), causing the immune system to no longer recognise them. This process, called “antigenic drift”, complicates the management of flu vaccination campaigns, determining the need to update vaccines annually and re-administer vaccines to the whole population [8]. On the other hand, there is another process called “antigenic shift”, consisting of major changes in HA and NA proteins of the virus that, although being less frequent, might lead to a potential pandemic effect [8]. This high virus variability creates a great challenge for public health in terms of both adequate and sufficient vaccine procurement and an efficient vaccination strategy. The immunisation drive is crucial if other cases are to be avoided, and we need to try every possibility of increasing the vaccination rates. For this purpose, it is essential to fight vaccine hesitancy, understanding the determinants of it, but also to provide the easiest and safest way to administer the vaccines. Moreover, it should be considered that influenza immunisation not only protects vaccinated individuals but provides some level of indirect protection, called “herd effects” or “herd immunity”. Even if indirect effects are assumed to provide a little additional benefit, it might make the difference when a large portion of the population is immunised [56]. In this perspective, and considering that intranasal spray administration is readily accepted, systematic delivery of influenza vaccine in all possible settings and with a large target population would greatly enhance the epidemic control [57].

As regards the safety of LAIV, healthy adults did not report a higher risk of adverse events when compared with placebo, opening up prospects for new targets. The LAIV appears to be manageable and particularly suitable for easy administration, being minimally invasive. Would this help increase vaccines acceptance? Indeed, appropriate communication of this information [58], and widespread dispersal of this knowledge among the general population, including social network channels [59], are essential. The flu vaccination coverage threshold is rarely achieved, but during the COVID-19 pandemic, great attention was also raised around flu vaccination [60], especially during the 2020/2021 flu vaccination campaign, while the COVID-19 vaccination was still not available. An extensive flu vaccination campaign was conducted in 2020 to better differentiate between flu and COVID-19 due to the similar symptomatology and consequently to be more sensitive in differential diagnoses among the two [61]. In this context, the flu vaccination request highly increased, obtaining a vaccination rate never reached before [62], but also causing procurement issues and vaccine shortages. In this case, would it be helpful to extend the use of LAIV to other groups, particularly healthy adults? Moreover, it should be considered that the LAIV spray vaccine can also be self-administered, reducing the efforts usually needed in planning, organising and implementing an injection vaccination campaign, and overstepping the fear of needles that is recurrent among the general population.

### Limits and Strengths

The main limitation of our study is relatively high heterogeneity in the characteristics of the included studies that allow us to only combine in meta-analysis a low number of studies or to quantitatively assess efficacy and safety of LAIV only for some serotypes, only in healthy adults, or not wholly exploring differences between LAIV compared with placebo and LAIV compared with IIV. Indeed, in our analysis, we previously combined studies that used IIV and placebo as control groups; however, in sensitivity analysis, we then only included studies with IIV as control or only including the placebo as control, based on the number of studies available (usually in case of less than three or four studies, meta-analysis is not recommended). Indeed, in most of our sensitivity analyses, very few studies were retrieved for each viral strain. Another potential limitation concerns the different populations included in our review. However, we believe that this element can represent both a limitation but also a strength. Having a so broad population can lead to heterogeneity. However, at the same time, it can allow us to explore different target populations simultaneously. Indeed, our study intended to stratify analysis based on a specific target population. Nevertheless, it was not possible due to the low number of studies retrieved for each specific population subgroup. Therefore, we could only stratify the analysis among healthy and immunocompromised subgroups of subjects. Moreover, since the results were expressed in several different ways in the original manuscript, we calculated the ES (expressed as OR) based on the reported events and total sample size in the two groups (intervention and control). This aspect might represent a limit since we use raw data without any adjustment for potential confounders. In other words, our calculated ES for each study and the overall result should be considered as a crude value. However, since the participants’ characteristics recruited in intervention groups and those in the control groups were similar, we believe that this does not affect the interpretation of our results. On the contrary, our study is the first systematic review with meta-analysis to assess the association between efficacy and safety of LAIV in target groups different from children older than 2 years [63,64,65]. In particular, we aimed to explore efficacy and safety in children below 2 years, adults, subjects at higher risk (as those immunocompromised, subjects with comorbidities, or pregnant/breastfeeding women). Moreover, this review has a systematic and comprehensive approach used to retrieve as much evidence as possible. Indeed, we consulted two different medical/scientific databases, and, in addition, we manually check the listed references. Furthermore, we conducted the review in agreement with the international guidelines and followed the approved checklist. In addition, our analyses showed no statistical heterogeneity (in most of the analyses, we found an I^2^ equal to zero), and no publication bias was detected by visual inspection of the funnel and performing the Egger’s regression test. Lastly, we performed both fixed and random effect models, allowing us a comparison among the two estimated ES values. However, since the I^2^ was equal to zero in most of the performed analyses, the two estimated ES were identical in almost all the analyses.

## 5. Conclusions

Reviews and meta-analyses can be very useful decision-making tools, providing evidence to instruct public health interventions and, in our case, to plan future vaccination campaigns.

Our review’s results supported the safety and efficacy of LAIV, even if our meta-analysis showed LAIV efficacy when compared against a placebo, becoming lower when compared to IIV. As mentioned above, we highlighted the scarcity of available studies and trials providing data for specific and vulnerable groups. From this perspective, we acknowledge our review as a starting point for future research pathways, and, due to the goof efficacy and very low invasiveness of LAIV, we believe that further analysis on efficacy, safety, and acceptance of this vaccine could address crucial public health issues, shaping current and future vaccination campaigns [66] and adjusting medical social measures to the context we live in.

## Figures and Tables

**Table 1:** Descriptive characteristics of included studies reported in alphabetical order.

Author, Year	Country	Study Design	Study Period	Sample Size	Population Characteristics	Comparison	Doses Administered and Scheme	Vaccine Composition	Outcomes	Methods	Funds	CoI
Ambrose, 2013 [32]	USA	RCT Allocation 2:1	1997/1998	I: 805 C:1420	Adults without high-risk diseases, 18–64 y, 58% F	Placebo	n.a.	n.a.	Safety	n.a.	yes	yes
Brady, 2018 [33]	USA	Double-blind, double-arm RCT Allocation 1:1	2011/2012 season, 2012/2013 season	I: 124 C: 124	Healthy breastfeeding women, 18–49 years, median age 31.4 y	IIV + intranasal placebo	n.a.	H1N1, H3N2, B	Efficacy, Safety	HAI assay, IgG and IgA ELISA	yes	yes
Carr, 2011 [34]	USA	RCT Allocation 1:1	14 October 2008 to 31 December 2008	I: 28 C: 27	Immunocompromised children with cancer 2–21 years, mean age 10.4 y for both groups 45.5% F	TIV	Children < 9 y two doses of vaccine, 28–42 days apart. Children ≥ 9 y single dose. 0.2 mL intranasally (0.1 mL per nostril)	H1N1, H3N2, B	Efficacy, Safety	HAI test	yes	yes
Forrest, 2011 [35]	Republic of South Africa	Prospective, randomised, open-label, multicenter trial. Allocation 1:1	March–November 2002	I: 1490 C: 1479	Community-dwelling adults, 60–95 y, mean age 69.2 ± 6.8 y; 62.1% F	TIV	Single dose, 0.2 mL	H1N1, H3N2, B	Efficacy, Safety	HAI assay, ELISPOT assay	yes	yes
Gruber, 1993 [36]	USA	Prospective, randomised, no placebo-controlled trial	Spring 1991	I: 98 C: 97	Healthy adults, ≤65 years Mean age: I: 36 y, C: 38 y. 2/3 female	Cold-adapted influenza A vaccine by nose drops	Single dose, five 0.1 mL sprays per nostril.	H1N1, H3N2	Efficacy, Safety	HAI assay	n.a.	n.a.
Hammitt, 2009 [37]	USA	Prospective, open-label, 2-arm, no placebo-controlled trial	October to November 2006	I: 10 C: 5	Healthy adults, 18–45 years	TIV	Single dose. Dosage not reported	H1N1, H3N2, B	Efficacy	HAI assay, IgG and IgA ELISA	yes	yes
King, 2000 [38]	USA	Double-blind RCT, stratified by HIV infection status Allocation 1:1	n.a.	HIV-infected adults: I:28, C:29	HIV group: mean age 40 y, 51% F;	Placebo	Single dose, 0.5 mL intranasal spray (0.25 mL per nostril)	H1N1, H3N2, B	Efficacy, Safety	HAI assay	yes	yes
				non-HIV-infected adults: I:27, C:27	non-HIV group: mean age 34 y, 65% F							
Kiseleva, 2020 [39]	Russia	Phase I, double-blind RCT Allocation 3:1	2018/2019 season	I: 30 C: 10	Healthy adults, 18–49 y, I: 32.6 ± 9.8 y; 40% F, C: 34.8 ± 9.3 y; 40% F	Placebo	Two doses 28-day apart, 0.5 mL	H7N9	Efficacy, Safety	HAI assay, MN assay, IgG and IgA ELISA	yes	no
Mallory, 2010 [40]	USA	Double-blind RCT Allocation 4:1	2009	I: 228 C: 55	Healthy adults, 18–49 y, I: 33.3 ± 9.2 y, 57.5% F, 82.9% white C: 34.1 ± 8.9 y, 55.0% F, 78.3% white	Placebo	Two doses 28 days apart, 0.5 mL	H1N1	Efficacy, Safety	HAI test	yes	yes
Manenti, 2017 [41]	Norway	Clinical trial	Winters 2012/2013	I: 15 C: 15	Healthy adults, I: mean age 34.6 y (19–59), 66% F C: mean age 44.9 y (26–64), 87% F	IIV	Single dose, 0.2 mL	H1N1, H3N2, B	Efficacy	HAI assay	n.a.	no
Nichol, 1999 [42]	USA	Double-blind RCT Allocation 2:1	September 1997 to March 1998	I: 3041 C: 1520	Healthy, working adults, 18–64 y, I: 38.3 ± 10.2, 54.7%F C: 38.2 ± 10, 54.3% F	Placebo	Single dose. Dosage not reported	H1N1, H3N2, B	Safety	n.a.	yes	yes
Phonrat, 2013 [43]	Thailand	Double-blind RCT Allocation 3:1	2009	I: 162 C: 56	Healthy adults, 19–75 y I (19–49 y group): 56.1% F I (50–75 y group): 91% F	Placebo	Two doses 21 days apart, 0.5 mL	H1N1	Efficacy, Safety	HAI assay, MN assay, IgG and IgA ELISA	yes	n.a.
Pitisuttithum, 2017 [44]	Thailand	Double-blind RCT Allocation 2:1	2013	I: 101 C: 51	Healthy adults, 18–49 y, 60.5% F	Placebo	Two doses 28 days apart, 0.5 mL	H5N2	Efficacy, Safety	HAI assay, MN assay, IgG and IgA ELISA	yes	no
Rudenko, 2014 [45]	Russia	Phase 1 double-blind RCT Allocation 3:1	April–July 2012	I: 30 C: 10	Healthy adults, 18–49 years I: mean age 30.1 y, 50% F C: mean age 38.5 y, 40% F	Placebo	Two doses 28 days apart, 0.5 mL	H7N3	Efficacy	HAI assay, MN assay, IgG and IgA ELISA	n.a.	n.a.
Rudenko, 2015 [46]	Russia	Phase 1 double-blind RCT	2012–2013	I: 30 C: 10	Healthy adults, 18–49 years old I: mean age 27.7 y C: mean age 29.2 y	Placebo	Two doses 4 weeks apart, 0.5 mL	H5N2	Efficacy, Safety	HAI assay, MN assay, IgG and IgA ELISA	yes	n.a.
Rudenko, 2016 [47]	Russia	Phase 1 double-blind RCT Allocation 3:1	October 2014 to April 2015	I: 29 C: 10	Healthy adults, 18–49 years, I: 27.6 ± 8.2, 50% F, 100% white C: 27.2 ± 8.8, 50%F, 100% white	Placebo	Two doses 28 days apart, 0.5 mL	H7N9	Efficacy	HAI assay, MN assay	yes	n.a.
Speroni, 2005 [48]	USA	Cross-sectional	November 2004 to March 2005	I: 63	I: average age 39.0, 81% F	IIV and unvaccinated	n.a.	n.a.	Safety	n.a.	n.a.	n.a.
				C1 = IIV: 201	C1: average age 49.0, 85.6% F							
				C2 = unvaccinated: 77	C2: average age 42.0, 83.0% F							
Treanor, 1999 [49]	USA	Double-blind RCT Allocation 1:1:1	December 1995 to January 1996	I: 36	Healthy adult volunteers,	Either CAIV-T with intramuscular placebo, or TIV with intranasal placebo, or intranasal and intramuscular placebo	Single dose, 0.5 mL	H3N2, H1N1, B	Efficacy, Safety	HAI assay	yes	n.a.
				C1 = TIV: 33	18–45 years,							
				C2 = placebo: 34	26% F							
van Voorthuizen, 1981 [50]	The Netherlands	Double-blind RCT Allocation 1:1	May 1979	I: 14 C: 14	Healthy volunteers, 19–28 years, 14.3% F	Placebo	Two doses 31 days apart; 0.5 mL (0.25 mL per nostril)	H1N1	Efficacy, Safety	HAI assay	n.a.	n.a.
Vesikari, 2008 [51]	Finland	Double-blind RCT Allocation 1:1	May–December 2002	In the 6-week to <16-week cohort: I: 31, C:28	In the 6-week to <16-week cohort: I: mean age 11.9 weeks, 58.1% F C: mean age 12.1 weeks, 53.6% F	Placebo	Two doses 35 (±7) days apart, 0.1 mL per nostril	H1N1, H3N2, B	Safety	n.a.	yes	n.a.
				In the 16-week to <24-week cohort: I: 30, C: 31	In the 16-week to <24-week cohort. I: mean age 20.1 weeks, 46.7% F C: mean age 19.9 weeks, 51.6% F							
Wang, 2009 [52]	USA	Longitudinal cohort study	1 September–30 April 2004, 2005, and 2006	2004/2005: I: 184,707 C1–TIV: 366,201 C2–unimmunised: 510,820 2005–2006: I: 143,054 C1–TIV: 626,478 C2–unimmunised: 271,732 2006–2007: I: 400,630 C1–TIV: 436,600 C2–unimmunised: 230,729	US military service members on active duty, 17–49 y, pregnant women excluded	TIV-immunised and unimmunised	Single dose. Dosage not reported	n.a.	Efficacy	The hospitalisation rate for pneumonia, influenza or ILI	n.a.	n.a.
White, 1976 [53]	UK	Clinical trial	January 1975	I: 51 C: 40	Volunteers among employees of British Leyland Limited (8% F)	Nose drops	Single dose, 0.5 mL (0.25 mL per nostril as nose drops in method A, or spray with three different spray devices in methods B, C, or D.	H3N2	Efficacy	HAI assay	n.a.	n.a.

C, comparison; CoI: Conflict of Interest; ELISA, enzyme-linked immunosorbent assay; HAI, hemagglutination inhibition assay; I, intervention; IIV, inactivated influenza vaccine; MN, microneutralisation assay; n.a., not applicable; TIV, trivalent inactivated vaccine; RCT, randomised clinical trial; UK, United Kingdom; USA, United States of America.

**Table 2:** Subgroup and sensitivity analysis assessing LAIV efficacy among immunocompromised subjects stratified by influenza serotype. ES: effect size, estimated as odds ratio.

Analysis	Model	Number of Studies Included	ES	95% CI	*p*-Value	Sample Size	I^2^	*p*-Value	Intercept	Tau (t)	*p*-Value
A/H1N1 (control group IIV)	Fixed	3	0.05	0.04–0.06	0.000	3099	91.77	0.000	3.28	1.43	0.388
	Random		0.29	0.02–4.10	0.361						
A/H1N1 (control group TIV or placebo)	Fixed	5	0.05	0.04–0.06	0.000	3397	86.82	0.000	1.58	1.02	0.383
	Random		0.15	0.02–1.08	0.059						
A/H3N2 (control group IIV)	Fixed	3	0.19	0.16–0.23	0.000	3241	72.80	0.025	−1.99	−1.63	0.350
	Random		0.13	0.06–0.28	0.000						
A/H3N2 (control group IIV or placebo)	Fixed	4	0.19	0.17–0.23	0.000	3292	69.9	0.021	−0.61	−0.42	0.713
	Random		0.16	0.07–0.35	0.000						
B (control group IIV)	Fixed	3	0.04	0.03–0.05	0.000	3242	45.95	0.157	0.59	0.41	0.752
	Random		0.05	0.01–0.18	0.000						
B (control group IIV or placebo)	Fixed	4	0.04	0.03–0.05	0.000	3294	44.27	0.146	0.89	1.01	0.418
	Random		0.06	0.02–0.22	0.000						

**Table 3:** Meta-analyses assessing LAIV safety among healthy and immunocompromised subjects stratified by adverse events following immunisation (AEFI). ES: effect size, reported as odds ratio.

Analysis	Model	Number of Studies Included	ES	95% CI	*p*-Value	Sample Size	I^2^	*p*-Value	Intercept	Tau (t)	*p*-Value
Fever (healthy, LAIV vs. placebo)	Fixed	6	0.59	0.32–1.09	0.092	2556	0.00	0.605	−1.18	−1.96	0.121
	Random		0.59	0.32–1.09	0.092						
Fever (immunocompromised, LAIV vs. placebo)	Fixed	4	0.52	0.21–1.26	0.145	226	0.00	0.735	−3.13	−1.93	0.193
	Random		0.52	0.21–1.26	0.145						
Fatigue/tiredness (healthy, LAIV vs. placebo)	Fixed	5	1.16	0.95–1.41	0.152	2604	0.00	0.651	−0.75	−2.43	0.093
	Random		1.16	0.95–1.41	0.152						
Myalgia (healthy, LAIV vs. placebo)	Fixed	4	1.17	0.93–1.46	0.171	2571	16.67	0.308	−0.57	−0.64	0.590
	Random		1.06	0.72–1.58	0.756						
Cough (healthy, LAIV vs. placebo)	Fixed	6	1.24	0.97–1.60	0.086	2643	39.70	0.141	−1.49	−4.84	0.008
	Random		0.87	0.47–1.62	0.666						
Cough (immunocompromised, LAIV vs. placebo)	Fixed	4	0.98	0.43–2.25	0.968	232	0.00	0.421	−1.52	−1.40	0.297
	Random		0.98	0.43–2.25	0.968						
Sore throat (healthy, LAIV vs. placebo)	Fixed	6	1.74	1.43–2.13	0.000	2643	41.99	0.125	−1.43	−4.44	0.011
	Random		1.12	0.62–2.03	0.703						
Headache (healthy, LAIV vs. placebo)	Fixed	5	1.03	0.87–1.23	0.696	2605	0.00	0.837	−0.26	−0.65	0.560
	Random		1.03	0.87–1.23	0.696						
Nasal Congestion (healthy, LAIV vs. placebo)	Fixed	6	2.33	1.34–4.04	0.003	446	0.00	0.768	0.03	0.05	0.959
	Random		2.33	1.34–4.04	0.003						
Rhinorrhea (healthy, LAIV vs. placebo)	Fixed	5	2.37	1.99–2.83	0.000	2579	51.83	0.081	−1.41	−2.69	0.074
	Random		1.55	0.80–3.02	0.194						

## Data Availability

All the data supporting reported results can be found either in the manuscript or the enclosed Supplementary Materials.

## Supplementary Materials

The following are available online at https://www.mdpi.com/article/10.3390/vaccines9090998/s1. Table S1: Search strategy algorithms for each database. Table S2: PICOS. Table S3: Distribution of 4-fold increase in antibody titer among intervention (I) and control (C) groups for each study, listed in alphabetical order. Table S4: (a) Distribution of adverse events following immunisations for intervention (I) and control (C) groups for each study, listed in alphabetical order (included in meta-analysis). (b) Distribution of adverse events following immunisations for intervention (I) and control (C) groups for each study, listed in alphabetical order (not included in meta-analysis). Table S5: Quality assessment of the included observational studies, in alphabetical order.
