# Supplementary material for: Safety and Efficacy of Spray Intranasal Live Attenuated Influenza Vaccine: Systematic Review and Meta-Analysis
Source: Vaccines (Basel). 2021 Sep 7;9(9):998. doi: 10.3390/vaccines9090998 (PMC8472940; doi:10.3390/vaccines9090998)
Supplement: Supplementary file 1 [file vaccines-09-00998-s001.zip › supplementary materials.v11.pdf]

Supplementary Table S1: Search strategy algorithms for each database.

SET	PubMed/MEDLINE	SET	Scopus
1	"Influenza vaccines" [MeSH]	1	(TITLE-ABS-KEY) Flu
2	"Flu" [Title/Abstract]	2	(TITLE-ABS-KEY) Fluenz
3	"Influenza" [Title/Abstract]	3	(TITLE-ABS-KEY) Fluenz tetra
4	"Fluenz" [Title/Abstract]	4	(TITLE-ABS-KEY) Flumist
5	"Fluenz tetra" [Title/Abstract]	5	(TITLE-ABS-KEY) Live-attenuated
6	"Flumist" [Title/Abstract]		
7	"Live attenuated" [Title/Abstract]		
8	"Live-attenuated" [Title/Abstract]		
9	"FluMist" [Supplementary Concept]		
10	Set 2-9 were combined with "OR"	6	Set 1-5 were combined with "OR"
11	"Nasal" [Title/Abstract]	7	(TITLE-ABS-KEY) Influenza AND Vaccine
12	"Intranasal" [Title/Abstract]	8	(TITLE-ABS-KEY) Nasal
13	"Intra-nasal" [Title/Abstract]	9	(TITLE-ABS-KEY) Intranasal
14	"Intra nasal" [Title/Abstract]	10	(TITLE-ABS-KEY) Intra-nasal
15	"Spray" [Title/Abstract]	11	(TITLE-ABS-KEY) Spray
16	Set 11-15 were combined with "OR"	12	Set 8-11 were combined with "OR"
17	"Mice" [Title/Abstract]	13	(TITLE-ABS-KEY) Mice
18	"Rodent" [Title/Abstract]	14	(TITLE-ABS-KEY) Rodent
19	"Rat" [Title/Abstract]	15	(TITLE-ABS-KEY) Rat
20	"Mouse" [Title/Abstract]	16	(TITLE-ABS-KEY) Mouse
21	"In vitro" [Title/Abstract]	17	(TITLE-ABS-KEY) In vitro
22	"In vivo" [Title/Abstract]	18	(TITLE-ABS-KEY) In vivo
23	"Equine" [Title/Abstract]	19	(TITLE-ABS-KEY) Equine
24	"Weanling" [Title/Abstract]	20	(TITLE-ABS-KEY) Weanling
25	"Pig" [Title/Abstract]	21	(TITLE-ABS-KEY) Pig
26	"Swine" [Title/Abstract]	22	(TITLE-ABS-KEY) Swine
27	"Pigs" [Title/Abstract]	23	(TITLE-ABS-KEY) Piglet
28	"Piglet" [Title/Abstract]	24	(TITLE-ABS-KEY) Chicken
29	"Piglets" [Title/Abstract]	25	Set 13-24 were combined with "OR"

30	"Chicken" [Title/Abstract]	26	Set 6, 7 and 12 were combined with "AND", set 25 was combined with "NOT"
31	"Chickens" [Title/Abstract]		
32	Set 17-31 were combined with "OR"		
33	Set 1,10 and 16 were combined with "AND", set 32 was combined with "NOT"		

Supplementary Table S2: PICOS.

Parameter	Description
**Population**	Inclusion: infants younger than 24 months, adults (≥18 years), patients with comorbidities and pregnant/breast feeding women<br>Exclusion: children/adolescents
**Intervention**	Inclusion: spray intranasal live-attenuated influenza vaccine<br>Exclusion: other types of influenza vaccines
**Comparison**	No influenza vaccine administration (placebo) or other types of influenza vaccines
**Outcome**	Inclusion: safety, efficacy, effectiveness<br>Exclusion: other outcomes
**Study design**	Inclusion: both randomized controlled trials and observational studies<br>Exclusion: studies not published as peer-reviewed, systematic review, meta-analysis, book, book chapter, thesis, protocol, no full-text papers (abstract, conference paper, letter, commentary, erratum, correction, editorial, note)

Supplementary Table S3. Distribution of 4-fold increase in antibody titer among intervention (I) and control (C) groups for each study, listed in alphabetical order.

Author, year [Ref]	Characteristics	I/C	Strain	Events/total sample (intervention group)	Events/total sample (control group)
Brady, 2018 [33]	Breastfeeding women	LAIV/IIV	A/H1N1	0/123	60/124
			A/H3N2	10/123	67/124
			B	0/123	29/124
Carr, 2011 [34]	Immunocompromised children with cancer	LAIV/TIV	A/H1N1	2/26	12/26
			A/H3N2	2/26	12/26
			B	1/26	3/26
Forrest, 2011 [35]	Elderly	LAIV/TIV	A/H1N1	123/1474	955/1469
			A/H3N2	296/1474	811/1468
			B	50/1474	723/1469
Gruber, 1993 [36]	Healthy adults	LAIV/nose drops	A/H1N1	39/99	31/99
			A/H3N2	40/99	30/99
King, 2000 [38]	HIV-seropositive	LAIV/placebo	A/H1N1	1/26	2/25
			A/H3N2	2/26	2/25
			B	0/26	1/25
	HIV-seronegative		A/H1N1	1/27	0/26
			A/H3N2	0/27	0/26
			B	1/27	0/26

Kiseleva, 2020 [39]	Healthy adults	LAIV/placebo	A/H7N9	16/30	0/8
Mallory, 2010 [40]	Healthy adults	LAIV/placebo	A/H1N1	32/189	3/42
Manenti, 2017 [41]	Healthy adults	LAIV/IIV	A/H3N2	6/15	13/15
Phonrat, 2013 [43]	Healthy adults 19-49 y	LAIV/placebo	A/H1N1	19/85	1/29
	Healthy adults 50-75 y		A/H1N1	19/77	1/27
Pitisuttithum, 2017 [44]	Healthy adults	LAIV/placebo	A/H5N2	13/100	0/49
Rudenko, 2014 [45]	Healthy adults	LAIV/placebo	A/H7N3	3/30	0/10
			A/H7N3	9/29	0/10
Rudenko, 2015 [46]	Healthy adults	LAIV/placebo	A/H5N2	11/29	0/10
			A/H7N9	19/29	0/10
Treanor, 1999 [49]	Healthy adults	CAIV-T/placebo	A/H1N1	7/30	5/32
			A/H3N2	10/30	2/33
			B	1/29	0/33
		CAIV-T/TIV	A/H1N1	7/30	30/33
			A/H3N2	10/30	25/33
			B	1/29	25/33
van Voorthuizen, 1981 [50]	Healthy adults	LAIV/placebo	A/H1N1	8/14	7/14
White, 1976 [53]	Healthy adults	LAIV/nose drops	A/H3N2	28/51	27/40

Supplementary Table S4 a. Distribution of Adverse Events Following Immunizations for intervention (I) and control (C) groups for each study, listed in alphabetical order (included in meta-analysis).

Author, year [Ref]	Characteristics	I/C	Fever		Fatigue/ Tiredness/Malaise		Myalgia		Cough		Sorethroat		Headache		Nasal congestion		Rhinorrhea	
			E/T I	E/T C	E/T I	E/T C	E/T I	E/T C	E/T I	E/T C	E/T I	E/T C	E/T I	E/T C	E/T I	E/T C	E/T I	E/T C
Ambrose, 2013 [32]		LAIV/p	8/805	17/1420	189/805	287/1420	138/805	203/1420	104/805	139/1420	217/805	229/1420	312/805	535/1420			380/805	372/1420
Brady, 2018 [33]		LAIV/TIV											53/123	48/124	55/123	26/124		
Carr, 2011 [34]	0-28 days from 1° dose	LAIV/TIV	1/28	4/27	6/28	5/27	0/28	3/27	3/28	5/27	6/28	3/27	5/28	4/27			12/28	9/27
Forrest, 2011[35]		LAIV/TIV	16/1390	20/1406			222/1455	220/1459	254/1452	179/1456	222/1452	148/1451	418/1456	375/1463	534/1457	352/1455		
King, 2000 [38]	NON-HIV	LAIV/p	0/27	3/27	4/27	6/27	4/27	6/27	3/27	7/27	6/27	6/27	12/27	11/27	21/27	12/27		
King, 2000 [38]	HIV	LAIV/p	2/28	3/29	10/28	6/29	10/28	6/29	11/28	8/29	7/28	2/29	11/28	9/29	17/28	9/29		
Kiseleva, 2020 [39]	1° dose	LAIV/p	0/30	1/8	1/30	0/8			0/30	1/8	0/30	0/8			1/30	0/8		
Pitisuttithum, 2017 [44]	1° dose	LAIV/p			0/101	0/51	3/101	0/51	5/101	5/51	6/101	3/51	9/101	2/51	9/101	2/51	10/101	8/51
Rudenko, 2016[47]	1° dose	LAIV/p	6/30	1/10					0/30	0/10	1/30	0/10	0/30	0/10	4/30	1/10	1/30	0/10
Speroni, 2005 [48]		LAIV/TIV	3/63	10/201	9/63	20/201	11/63	30/201	13/63	10/201	10/63	12/201	15/63	25/201	24/63	14/201	18/63	12/201
Speroni, 2005 [48]		LAIV/Unvaccinated	3/63	9/77	9/63	15/77	11/63	19/77	13/63	14/77	10/63	16/77	15/63	22/77	24/63	18/77	18/63	18/77
Treanor, 1999 [49]		CAIV-T/TIV	2/36	0/33														
Treanor, 1999 [49]		CAIV-T/p	2/36	3/34														

vanVoorthuizen, 1981 [50]		LAIV/p							5/14	4/14	5/14	3/14
Vesikari, 2008 [51]	6-to<16week of life	CAIV-T/p	3/31	3/28	7/31	1/28	0/31	2/28			2/31	5/28
Vesikari, 2008 [51]	16-to<24week of life	CAIV-T/p	5/30	5/31	3/30	4/31	1/30	1/31			5/30	5/31

continue

Supplementary Table S4 b. Distribution of Adverse Events Following Immunizations for intervention (I) and control (C) groups for each study, listed in alphabetical order (not included in meta-analysis).

Author, year [Ref]	Characteristics	Abdominal pain		Chills		Decreased activity		Decreased appetite		Diarrhoea		Herpes		Irritability		Nusea/ vomiting		Rash		Conjunctivitis		URI		Pain at Injection Site	
		E/T I	E/T C	E/T I	E/T C	E/T I	E/T C	E/T I	E/T C	E/T I	E/T C	E/T I	E/T C	E/T I	E/T C	E/T I	E/T C	E/T I	E/T C	E/T I	E/T C	E/T I	E/T C	E/T I	E/T C
Ambrose, 2013 [32]																									
Brady, 2018 [33]																						13/123	13/124	9/123	67/124
Carr, 2011 [34]	0-28 days from 1° dose			3/28	2/27									3/28	4/27	8/28	6/27								
Forrest, 2011 [35]				74/1437	63/1449	271/1447	225/1450	104/1439	76/1446							29/1429	33/1449								
King, 2000 [38]	NON-HIV	1/27	3/27					3/27	5/27							1/27	3/27								
King, 2000 [38]	HIV	2/28	1/29					3/28	3/29							4/28	5/29								
Kiseleva, 2020 [39]	1°dose											0/30	1/8												
Pitisuttithum, 2017 [44]	1°dose			0/101	0/51					1/101	0/51					3/101	0/51	2/101	0/51						
Rudenko, 2016 [47]																									
Speroni, 2005 [48]																									

Speroni, 2005 [48]								
Treanor, 1999 [49]								
Treanor, 1999 [49]								
van Voorthuizen, 1981 [50]								
Vesikari, 2008 [51]	6-to <16week of life		0/31	1/28		2/31	3/28	
Vesikari, 2008 [51]	16-to <24week of life		1/30	2/31		2/30	1/31	

C: control; E: events; I: intervention; p: placebo; T: total; URI: Upper Respiratory Infections

Supplementary Table S5: Quality assessment of the included observational studies, in alphabetical order.

**Cross-sectional/ Cohort studies, quality assessed by NOS**										
**Author year [Ref]**	**Selection**				**Comparability**		**Outcome/Exposure^**			**Tot/9**
	**Item 1**	**Item 2**	**Item 3**	**Item 4**	**Item 5a**	**Item 5b**	**Item 6**	**Item 7**	**Item 8**	
Speroni, 2005 [48]	*	*	-	*	*	-	-	*	*	6/9
Wang, 2009 [52]	*	*	*	*	*	*	*	*	*	9/9
